# Impacts of Saharan Dust Intrusions on Bacterial Communities of the Low Troposphere

**DOI:** 10.1038/s41598-020-63797-9

**Published:** 2020-04-22

**Authors:** Elena González-Toril, Susana Osuna, Daniel Viúdez-Moreiras, Ivan Navarro-Cid, Silvia Díaz del Toro, Suthyvann Sor, Rafael Bardera, Fernando Puente-Sánchez, Graciela de Diego-Castilla, Ángeles Aguilera

**Affiliations:** 1 Centro de Astrobiología (CSIC-INTA). Carretera de Ajalvir Km 4, Torrejón de Ardoz, 28850 Madrid, Spain; 2 Department of Genetics, Physiology and Microbiology. Biology Faculty. C/José Antonio Novais, 12, Universidad Complutense de Madrid (UCM), 28040 Madrid, Spain; 3 Aerodinamic Department (INTA). Carretera de Ajalvir Km 4, Torrejón de Ardoz, 28850 Madrid, Spain; 4 Systems Biology Program. Centro Nacional de Biotecnología. C/ Darwin n° 3, Campus de Cantoblanco, 28049 Madrid, Spain

**Keywords:** Biodiversity, Microbial ecology, Air microbiology

## Abstract

We have analyzed the bacterial community of a large Saharan dust event in the Iberian Peninsula and, for the first time, we offer new insights regarding the bacterial distribution at different altitudes of the lower troposphere and the replacement of the microbial airborne structure as the dust event receeds. Samples from different open-air altitudes (surface, 100 m and 3 km), were obtained onboard the National Institute for Aerospace Technology (INTA) C-212 aircrafts. Samples were collected during dust and dust-free air masses as well two weeks after the dust event. Samples related in height or time scale seems to show more similar community composition patterns compared with unrelated samples. The most abundant bacterial species during the dust event, grouped in three different phyla: (a) *Proteobacteria: Rhizobiales*, *Sphingomonadales, Rhodobacterales*, (b) *Actinobacteria: Geodermatophilaceae*; (c) *Firmicutes: Bacillaceae*. Most of these taxa are well known for being extremely stress-resistant. After the dust intrusion, *Rhizobium* was the most abundant genus, (40–90% total sequences). Samples taken during the flights carried out 15 days after the dust event were much more similar to the dust event samples compared with the remaining samples. In this case, *Brevundimonas*, and *Methylobacterium* as well as *Cupriavidus* and *Mesorizobium* were the most abundant genera.

## Introduction

Airborne microbes are ubiquitous in the atmosphere^1^ and are thought to play important roles in meteorological processes (i.e. clouds and snow formation or precipitation patterns alterations)^2,3^, as well as in the long-range dispersal of plant and livestock pathogens^4,5^ and in the maintenance of the diversity in aquatic systems^6^. Likewise, airborne bacteria can have important effects on human health, producing allergic asthma and seasonal allergies, and could interfere in the productivity of managed and natural ecosystems^7^. Global abundance of aerial microorganisms has been estimated, based on data from terrestrial environments, to range between 10^4^ to 10^6^ m^−3^ ^8^. However, more recent studies, incorporating direct counting by microscopy or quantitative PCR^9^, have yielded, more accurate, higher estimates of the abundance of airborne microbes and seasonal patterns^10^. Additionally, the atmosphere is one of the most extreme environments and microorganisms inhabiting in the troposphere are exposed to higher UV radiation, desiccation, cold temperatures and nutrient deprivation than in other environments^11,12^.

Several studies have also demonstrated the potential for microorganisms to be transported over long distances through the atmosphere, associated to desert dust, as a route for the colonization of new habitats^13,14^. Thus, desert dust clouds may serve not only as a source of nutrients for terrestrial plants and primary producers in nutrient depleted oceanic waters^15,16^, but may also serve as a vehicle for global transport of microorganisms, including pathogens^13,17^.

The Sahara-Sahel regions of North Africa are the dominant sources of aerosolized soil dust in the North Hemisphere atmosphere (50 to 75% of the current estimate), contributing as much as one billion metric tons of dust per year to the atmosphere^13,18^. Large dust storm events that originate from North Africa are capable of continent-wide, transoceanic, and global dispersion^13,17^ and frequently affect air quality in Africa, the Middle East, Europe, Asia, the Caribbean, and the Americas^15,16,18–21^. However, the southern Mediterranean is the most influenced areas by atmospheric intrusions of Saharan dust^18,22^. This intrusions account for ~50% of global dust production^23^. It has been estimated that 80–120 tn of dust per year are carried across the Mediterranean towards Europe, with dust clouds recorded to reaching an altitude of up to 8 km the Mediterranean basin^24–26^.

Despite the importance of the aerial microbial dispersion phenomena, studies dealing with the microbial ecology associated to African dust are very limited, being almost nonexistent in southern European areas. African dust-associated microbes has been explored after transatlantic long-distance transport^17,21,27–29^, after dust deposition in snow (Dolomite Alps)^30^, in high mountain lakes (Pyrenees)^6^, over the Turkish Mediterranean coastline^31,32^, Greece^33^, Corsica and Sardinia (Italy)^34^ and in the southern Iberian Peninsula^35^. These studies conclude that, although there is higher diversity in the airborne microbial composition during dust events, the amounts and the specific microbes associated remain topics of debate.

Furthermore, most studies to date on the atmospheric microbiome are restricted to samples collected near the Earth’s surface (e.g., top of mountains (ca. 2 km) or buildings). The tropospheric microbial ecology at high altitudes and in open air masses, where long-range atmospheric transport is more efficient, remains poorly characterized. This fact is mostly due to difficulties associated with obtaining enough biomass for analysis, not to mention the difficulty to access to aerial platforms (i.e. aircrafts) to sampling the air from the microbiological point of view. In this regard, to our knowledge, there are only four references regarding microbial ecology in the troposphere by using aircrafts, one related to tropical storms^36^, another to the airborne bacteria in the lower stratosphere^31^ and two reporting changes in the microbial atmospheric composition during Asian dust events in Japan^32,37^. Increasing our knowledge of this field will also contribute to improved models of microbial dispersal and microbial biogeography^36^.

In order to reduce these knowledge deficiencies, we obtained several open air high-altitude samples (up to ∼3 km) by using aircrafts during a severe Saharan dust intrusion in the Iberian Peninsula in the winter of 2017. Due to the advances in culture-independent techniques such as massive Illumina sequencing technologies, we were able to study the changes in the airborne bacterial community during and after this dust event at different altitudes. Airborne bacterial diversity was estimated based on their 16S rRNA gene sequences on both, Saharan dust days and non-Saharan dust days. Our results provide new insights into the species composition, relative abundance and dynamics of the bacterial and dynamics of the bacterial communities of the lower troposphere and, for the first time, the tropospheric influences of an African dust event.

## Materials and methods

### Sampling locations and collection

**Figure 1:**
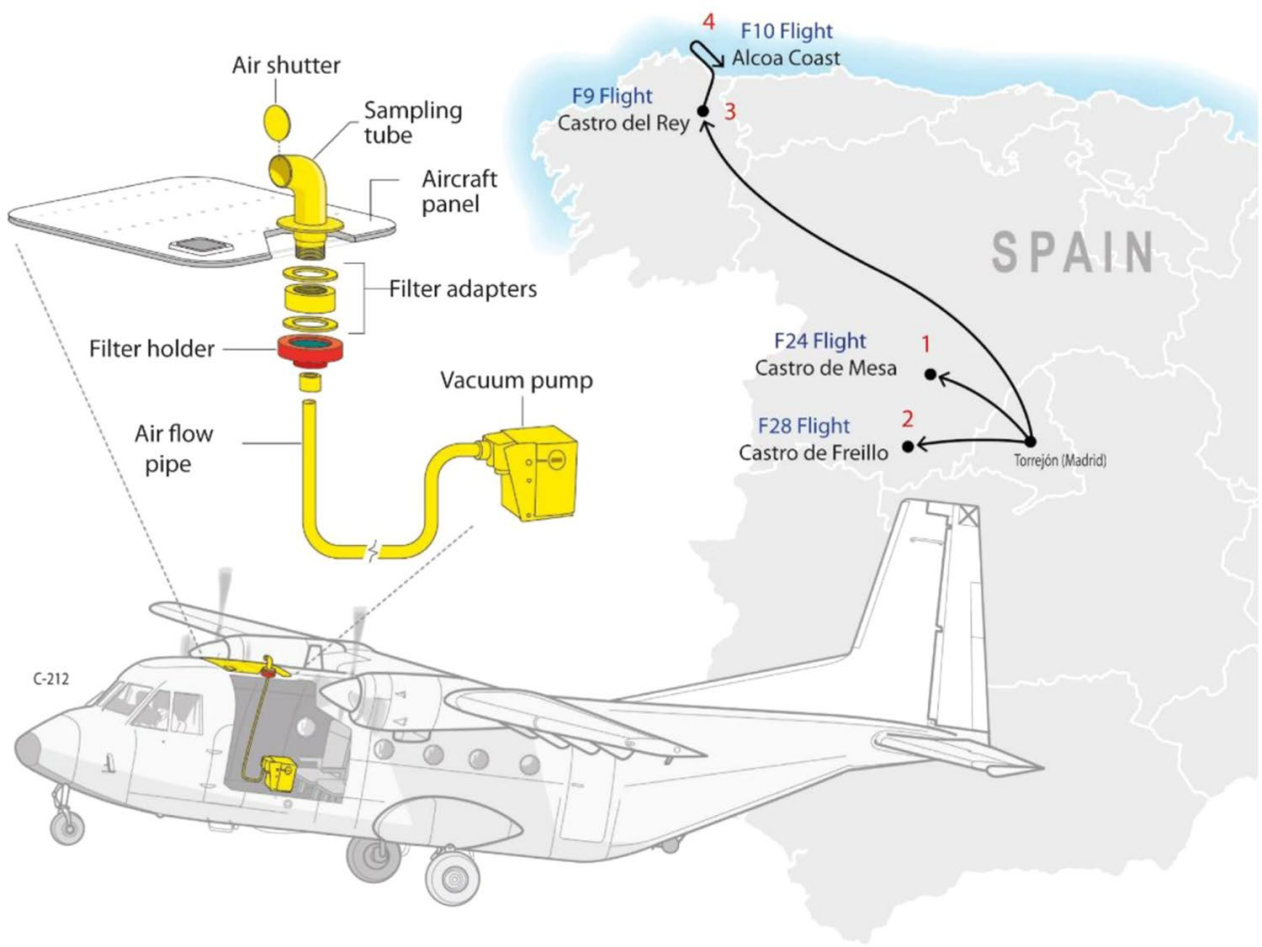
Schematic diagram of the atmospheric bioaerosol sampling device used in the C-212 airplane, as well as the approximated trajectories of the four flights carried out.

Samples were obtained onboard the National Institute of Aerospace Technology (INTA) C-212 aircraft platform. Air outside of the airplane was collected using the onboard sampler. The inlet was inserted into the air intake, which opens at the leading edge of the airplane, to avoid boundary effects. Bioaerosols were collected on glass fiber filter PM_10_ (X100, EPM2000, 47 mm in diameter, Whatman) by filtering air from outside the aircraft using a vacuum pump (Fig. 1). Glass fiber filters were previously sterilized in a dry oven (400 °C, 4 hours) in order to remove all possible DNA contaminates. Before every sampling flight, the filter adapters, the filter holder and the air shutter were submerged in isopropyl alcohol for, at least 4 hr in order to be sterilized. After that, it was dried in a laminar flow hood with the UV connected. At the same time, surface air samples were collected using the same sampling device, in the same approximate area in which the airplane was sampling and during the same time (60 min). A subsample of ca. 1cm^2^ of each filter was cut with a sterile scalpel and stored at 4 °C until electronic microscopy observation were carried out. Remaining filters were kept at −20 °C until DNA extraction.

To account for possible contamination associated with sample processing, appropriate negative controls were added: (i) a blank control for detecting molecular kit contaminates^38,39^ (ii) a blank glass fiber filter PM10 (X100, EPM2000, 47 mm in diameter, Whatman) previously sterilized in a dry oven (400 °C, 4 hours) (iii) swabs in 5 ml tubes wetted with PBS (Phosphate Buffered Saline, pH 7.4) for detecting contaminates on the inlet surface previously rinsed with isopropyl alcohol. These negative controls were treated exactly the same as all samples through the entire experiments process, including amplification, sequencing and OTU detection.

**Figure 2:**
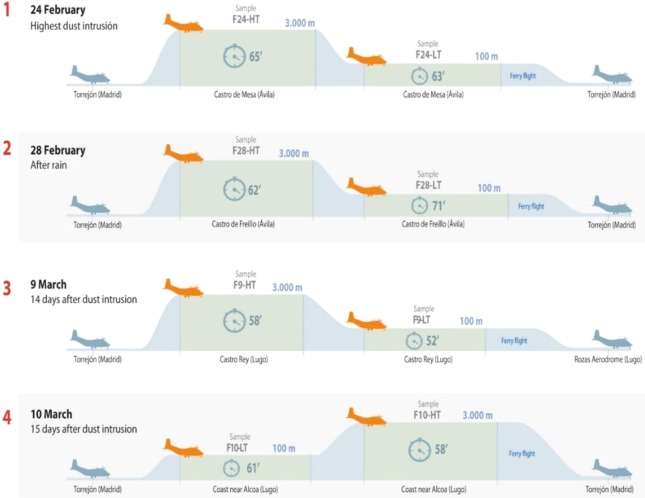
Flight profiles and sampling information (altitude, sampling time, locations).

The occurrence of atmospheric Saharan dust and its drift were confirmed by LIDAR (Light Detection and Ranging) data from the NOAA and Saharan dust intrusion from Spanish Meteorological Agency (AEMET, www. aemet.es). The geographic origin of African dust was determined by back-trajectory analysis (http://ready.arl.noaa.gov/HYSPLIT.php). The flights covered a total period of about 4 weeks (February 23, 2017–March 10, 2017). Air samples were collected from 23 and 24 February 2017 (dust severe conditions), 28 February 2017 (3 days after the end of the dust intrusion and after a heavy rain), and 9–10 March 2017 (15 days after the end of the dust event) (Table 1). The airplane took off from Torrejón Military Aerial Base (Madrid) and started collection of samples in the air of Avila (80 km NW Madrid), and Lugo (400 km NW Madrid). Samples from ∼100 m and ∼3,000 m were taken during the four flights, in addition to the surface samples mentioned previously (Table 1, Fig. 2). During each flight, biomass from atmospheric air was collected over a period of 1 h on average per sample. In total, over 15,000 l were filtered and thus, the samples were presumably representative of the air masses sampled. During sampling, we recorded the time, altitude, longitude and latitude, visibility, aircraft heading and course, airspeed and ground speed.

**Table 1:** Averaged sampling data.

Date	Sample*	Local time (h)	Sampling area	LAT (deg)	LONG (deg)	Altitude (m)	Meteorology
23/02/2017	SU-23F	11:30	Avila (NW Spain)	40.52	−4.38	1.5	High Dust
24/02/2017	SU-24F	11:00	Avila (NW Spain)	40.52	−4.38	1.5	High Dust
24/02/2017	F24-HT	11:0	Avila (NW Spain)	40.52	−4.38	2740**	High Dust
24/02/2017	F24-LT	12:00	Avila (NW Spain)	40.72	−4.94	100	High Dust
28/02/2017	SU-28F	11:00	Avila (NW Spain)	40.30	−4.60	1.5	After rain
28/02/2017	F28-HT	11:00	Avila (NW Spain)	40.33	−4.60	2740**	After rain
28/02/2017	F28-LT	13:00	Avila (NW Spain)	40.72	−4.94	120	After rain
09/03/2017	SU-9M	13:00	Lugo (NW Spain)	42.85	−6.52	1.5	Pristine
09/03/2017	F9-HT	13:00	Lugo (NW Spain)	42.85	−6.52	3040**	Pristine
09/03/2017	F9-LT	14:00	Lugo (NW Spain)	43.10	−7.47	160	Pristine
10/03/2017	F10-HT	11:00	Lugo (NW Spain)	43.57	−7.28	3040**	Pristine
10/03/2017	F10-LT	12:00	Lugo (NW Spain)	43.57	−7.28	100	Pristine

*Sample: (SU) Surface; (F) Flight; (HT) High Troposphere flight; (LT) Low Troposphere flight.

**Altitude over the sea level.

### Scanning electron microscopy (SEM) and energy dispersive x-ray spectroscopy (EDS)

Subsamples of each filter were examined in a Jeol 5600LV scanning electron microscope (SEM) equipped with a backscattered electron (BSE) detector and an INCA Oxford X-ray energy dispersive spectroscopy (EDS) microanalytical system. The size, morphology, and chemical composition of individual particles (ca.15–20 particles per sample) as well as areas of ca. 400 μm^2^ of each sample was evaluated. Conditions were set to 15 kV accelerating voltage and 100 s of effective counting time. Matrix corrections were made following the standard procedures using a combination of silicate, oxides, and pure metals (wollastonite for Ca and Si, jadeite for Na, orthoclase for K, corundum for Al, periclase for Mg, metallic Fe and Ti for Fe and Ti).

### Model for back-trajectory simulations

The back-trajectories of air masses were computed using the HYbrid Single-Particle Lagrangian Integrated Trajectory (HYSPLIT) model^40^, accessed via web-based Real-time Environmental Applications and Display sYstem (READY) and developed by the National Oceanic and Atmospheric Administration (NOAA) Air Resources Laboratory^41^. Thus, the Global Data Assimilation System (GDAS) was input to the HYSPLIT model in order to compute 3-days back-trajectories for air masses, taking into account each sampling location and time (Suplementary Table S1). Trajectories were computed during a time period *t-∆t*, where *t* is a particular sampling time and *∆t* is selected to be equal to 36 h, in time intervals of 6 h, in order to get insights at possible changes in the atmospheric patterns (e.g. winds) just previous to the sampling time, which could imply more than one predominant air mass trajectory influencing the concentrations within a particular parcel sampled. Altitudes around the sampling altitude were also derived  for sensitivity analysis, showing similar results. In addition, the concentration of Saharan dust in the atmosphere, particularly during the Saharan dust intrusion, was obtained by means of HYSPLIT in its dispersion model approach, accessed by the Barcelona Supercomputing Center.

### DNA extraction, amplicons preparation and Illumina sequencing

Filters were cut into small pieces with a sterile scalpel, and introduced into a Lysing Matrix Tube from FastDNA SPIN Kit for Soil (MP Biomedicals, LLC). To disrupt cells, three 30-s pulses of the FastPrep-2 5 G Instrument (MP Biomedicals, LLC) at speed 4.0 were performed. Genomic DNA extraction and elution was done according to the manufacturer’s instructions and purified DNAs were quantified by Quant-iT Pico-Green dsDNA Assay Kit (Invitrogen).

DNA extracted was used in a first PCR of 27 cycles with Q5 Hot Start High-Fidelity DNA Polymerase (New England Biolabs) in the presence of 100 nM primers for 16 S amplification, V3V4-CS1 (S-D-Bact-0341-b-S-17) (5′ACACTGACGACATGGTTCTACACCTACGGGNG GCWGCAG-3′) and V3V4-CS2 (S-D-Bact-0785-a-A-21) (5′-TACGGTAGCAGAGACTTG G T CTGACTACHVGGGTATCTAATCC-3′), these primers amplify the V3-V4 region of 16 S^42^. Each sample was amplified and, after the first PCR, a second PCR of 14 cycles was performed with Q5 Hot Start High-Fidelity DNA Polymerase (New England Biolabs) in the presence of 400 nM of primers (5′ AATGATACGGCGACCACCGAGATCTACACTGACGACATGGTT C TACA-3′ and 5′-CAAGCAGAAGACGGCATACGAG AT-[barcode]-TACGGTAGCAGAGAC TTGGT CT-3′) of the Access Array Barcode Library for Illumina Sequencers (Fluidigm). The finally obtained amplicons were validated and quantified by an Agilent 2100 Bioanalyzer using DNA7500 chips, and an equimolecular pool of these amplicons was purified by agarose gel electrophoresis to eliminate primers/dimers. This purified pool was titrated by quantitative PCR using the “Kapa-SYBR FAST qPCR kit forLightCycler480” and a reference standard for quantification. Finally, the pool of amplicons was denatured prior to be seeded on a flowcell at a density of 10pM, where clusters were formed and sequenced using a “MiSeq Reagent Kit v3”, in a 2 × 300 pair-end sequencing run on a MiSeq sequencer.

### OTU detection and classification

Sequence processing and analyses procedures were carried out using Mothur v.1.38.1^43^. Sequences were pre-processed by strictly following the standard operating procedure (MiSeq SOP)^44^. Pre-processed sequences, aligned and normalized, were used to generate an uncorrected pairwise distance matrix, which was then used to cluster the sequences using the furthest neighbor algorithm and to detect OTUs at 0.03 distance level. OTUs detected at a genetic distance of 0.03 were classified by comparing a representative sequence from each cluster against the Silva (v123) reference database^45^, using BLAST to identify similar sequences^46^ and the k-Nearest Neighbor algorithm to determine the consensus taxonomy from the 10 most similar sequences in the database. Sequences were classified according to the RDP6 taxonomy scheme^47^. Chao 1 estimator, Shannon and Simpson indices were calculated to predict community richness using PAST software^48^. The sequences reads in the negative controls were compared with the sequences read in the samples by BLAST. All sample sequences that showed a similarity ≥98% with the control sequences were eliminated prior taxonomic OTU assignment.

### Statistical analysis

Principal Component Analyses (PCA) on the relative proportion of OTUs (at a 0.03 distance) among the samples were obtained. Using the three components as new variables, hierarchical dendrograms by Euclidean squared distance method were also calculated (IBM SPSS Statistics 23 package)^49^. Shannon and Simpson diversity indices and number of OTUs (at 0.03 distance) as well as Chao1 index were used as biological dataset (CANODRAW 4.0 software was used for graphical presentation).

## Results and Discussion

Significant intensification of Sahara dust intrusions has been noticed during the last few decades^50^. These intrusions, and the subsequent dust deposition, are relevant for nutrient dispersal and soil development^51,52^, as well as for the introduction and dispersal of external microorganisms, including pathogens, into ecosystems and populations^17,30,53^. Despite the relevance of these phenomena, there are few studies analyzing variations on the atmospheric microbial diversity as a result of Sahara dust intrusions in Southern Europe^34,35,54,55^. Here, we analyzed the microbial load of one of the largest winter-occurring Saharan dust events in the Iberian Peninsula and, for the first time, we offer new insights into bacterial distribution at different altitudes of the lower troposphere, as well as the replacement of the native microbial airborne structure as the dust event disappears.

### Samples collected and microscopic analysis

The sampling campaign operated three continental flights within the Iberian Peninsula (flights from Madrid to Avila and Lugo (NW Spain)) and one flight off the coast in the north (Fig. 1) during which samples were taken. Macroscopic differences among the filters in terms of dust/particles recovery were clearly distinguished, showing lower coloration as the altitude increases (Suplementary Fig. S1). SEM analysis showed that the samples collected during the dust event were a collection of loose grains to which small particles adhered (Suplementary Fig. S2). Under these *in situ* conditions, it was difficult to distinguish microbes from clay particles, diatom fragments and other adhering material. As expected, samples from the other flights showed lower amounts of dust particles. Nevertheless, obvious cellular structures were visible in all samples analyzed. The most frequent images clearly shows rounded individual cells-like particles of ca. 6–10 μm, as well as cells forming clusters of variable shapes and sizes. Cells and clusters are always covered by an irregular coat of extracellular polymeric substances (EPSs). Fragments of diatom shells could be also seen.

Dust particles showed irregular morphology and great size variability (<10–30 μm) (Suplementary Fig. S2A, B). Elemental composition determination by SEM-EDS analysis showed that particles collected during the Saharan intrusion were composed mainly of Si (ca. 50 Wt%), Al and Fe (ca. 10 Wt%), as well as some trace elements such as Ti, Cr, Pb, Sr, Ba, Zr, Ag and Mn (Suplementary Fig. S3). These trace elements were not found in the remaining samples. Particles from flights F28, F9 and F10, were mainly composed by NaCl, although F28 presented a lesser degree of ions related to salt, and a major influence from soil.

Air mass back trajectories pointed to the Saharan/Hoggar Massif area as the main origin of this dust episode. In this region, the local geology is dominated by carbonate depocenter^56^. Soils from this desert area show high chemical diversity (i.e. hornblende and alkali feldspar, kaolinite, illite or montmorillonite)^56^. The results of EDS analysis showed a higher presence of Si, Mg, K, Mn and some trace elements such as Ti, V, Sr, Ba, Zr, Ni, Cr, Mo, Cu, Ag and Pb during Saharan dust intrusion. Average concentrations during non-Saharan dust periods (Atlantic air mass), showed higher amounts of Fe, Al, Na and Cl and none of the trace elements present during the dust event.

The major elements identified in the filters during the dust event showed the representative composition of particulate matter from the Saharan desert^57^. However, trace element composition present in the samples are not characteristic of the area and could be related to industrial emissions, Thus, the main sources of Ti, V, Mo, Cr and Pb are oil refineries, power plants, and phosphate-based fertilizer plants located in North Africa and Southern Spain^58^. Therefore, given the direction of the air masses and the composition of the particulate matter in the samples during the dust event, we suggest that the dust collected there came mainly from the Sahara, although some additional industrial emission material was collected on its way from the Sahara to Spain. This fact has also been reported by Sánchez de la Campa *et al*. (2013) in the only microbiological study related to Sahara dust intrusions in Spain conducted to date.

### Trayectory analysis

**Figure 3:**
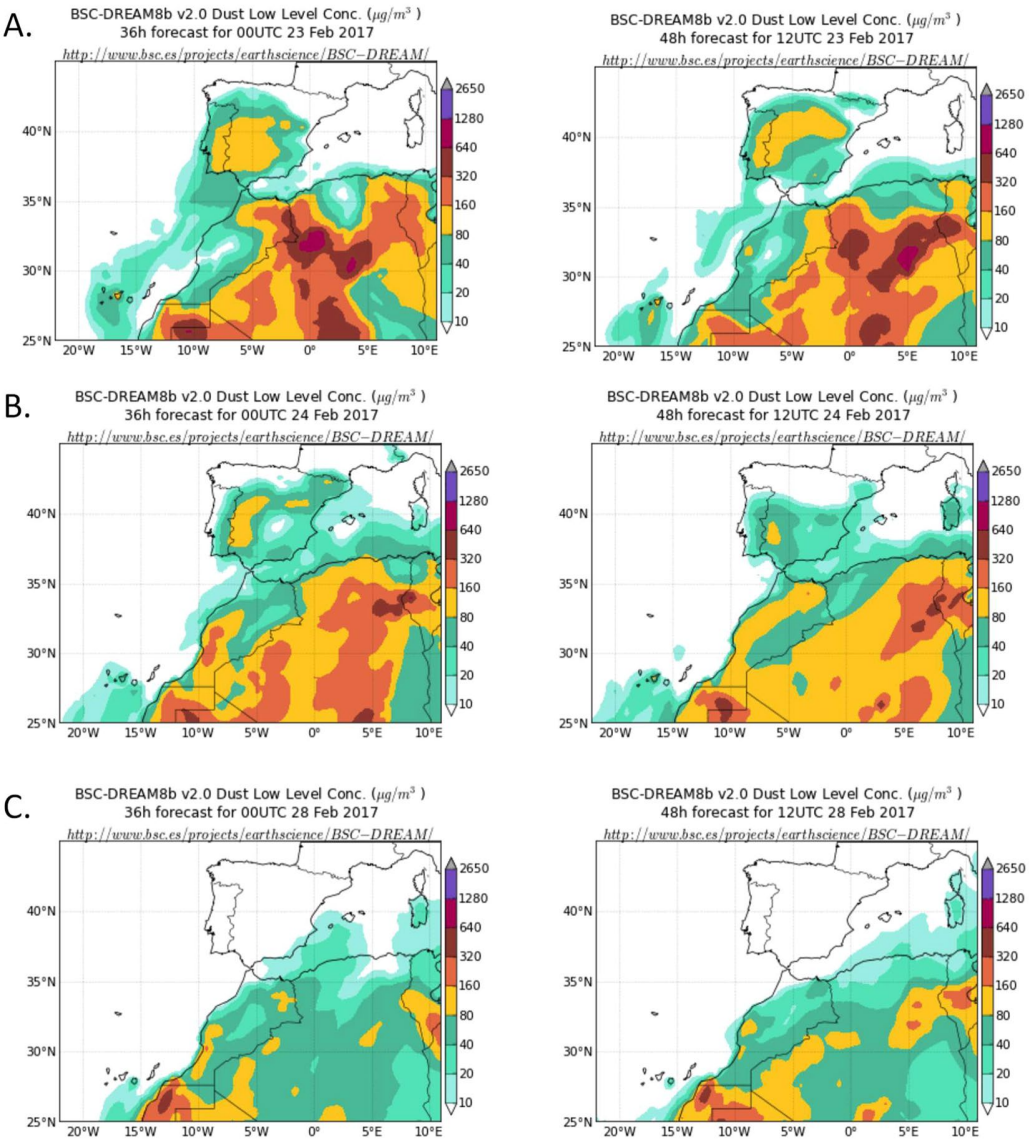
Dust concentration (μg/m3) predicted by model BSC-DREAM8b v2.0 for (**A**) 23 February 2017 at 00 UTC (left) and 12 UTC (right). (**B**) 24 February 2017 at 00 UTC (left) and 12 UTC (right). (**C**) 28 February 2017 at 00 UTC (left) and 12 UTC (right). On March 9th and 10th, dust concentration data for the Iberian Peninsula was very similar to that of February 28th (data not shown), predicted by model BSC-DREAM8b v2.0, Barcelona Supercomputing Center.

Dust concentration from 23 February to 10 March 2017, in 2-day intervals is summarized in Fig. 3. As observed, this period was characterized by a Saharan dust intrusion in the Iberian Peninsula, which was particularly strong between 23–24 February. After this date, the wind pattern above the Iberian Peninsula had changed to predominant north-northwesterly winds coupled with precipitation during these days, that favored dust deposition in the Iberian Peninsula (see the dust concentration decrease after 24 February in Fig. 3).

**Figure 4:**
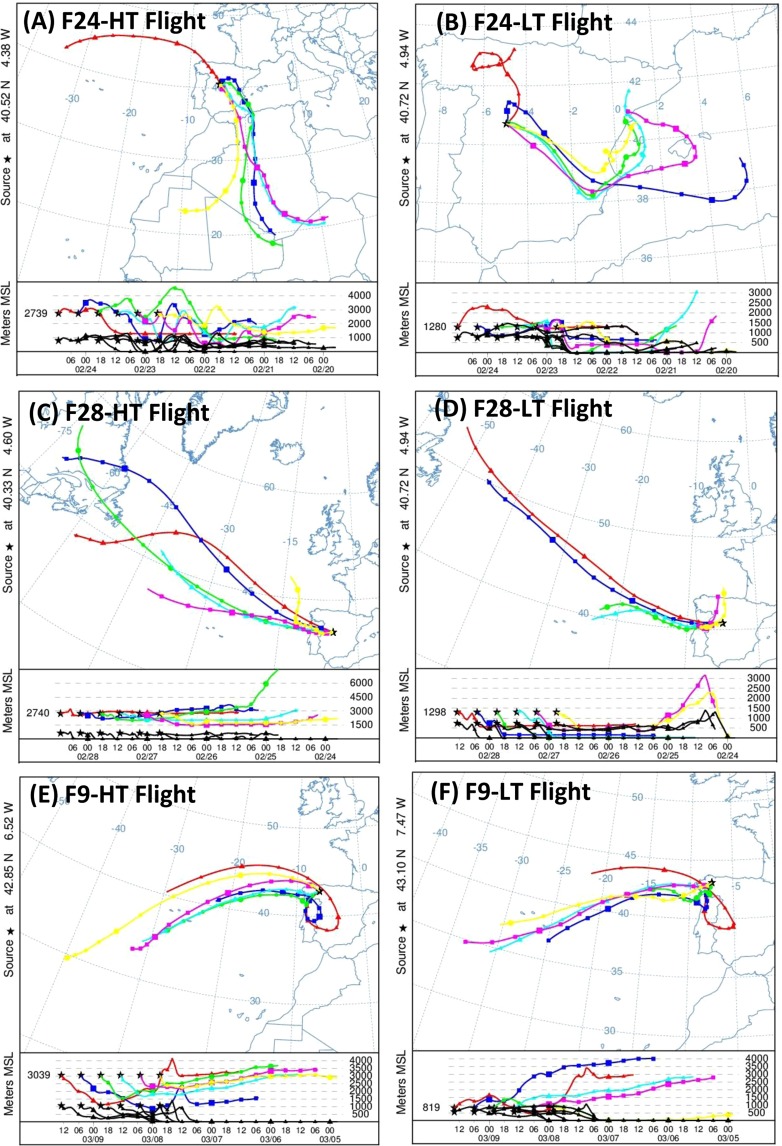
Three-days back-trajectories computed by HYSPLIT for the different sampling area flights. Air mass trajectories within the 48h-interval previous to the sampling time. (**A**) F24-HT flight (3,000 m altitude); (**B**) F24-LT flight (100 m altitude); (**C**) F28-HT flight (3,000 m altitude); (**D**) F28-LT flight (100 m altitude); (**E**) F9-HT flight (3,000 m altitude); (**F**) F9-LT flight (100 m altitude). F10 flights presented similar trajectories than F9 flights.

Air masses sampled on each flight were found to have a different origin on average, based on HYSPLIT simulations. F24-HT, sampled on Feb 24 at ~3,000 m, showed air masses coming both from North Africa and the Atlantic Ocean (Fig. 4A). Thus, those coming from North Africa (Feb 22–23) experienced strong vertical mixing with different atmospheric layers, including the Saharan Planetary boundary layer (PBL)^33,59^. On Feb 24, the wind pattern changed above the Iberian Peninsula, rotating to north-northwesterly winds; as a result, the origin of air masses was originated in the Atlantic Ocean for this particular day. Therefore, the resulting Saharan dust remaining in the atmosphere is expected to be the main source contributing to the sample. F24-LT, roughly sampled at the same time and location as F24-HT, but at ~100 m above the ground level (Suplementary Table S1), showed a similar change in the air mass pattern. On Feb 22 and Feb 23 predominant air masses originated from the eastern part of the Iberian Peninsula and Spain’s Mediterranean coast, with strong interaction within the PBL above the southeast and center of the Iberian Peninsula. On the contrary, predominant air masses on Feb 24 came from the north of Iberian Peninsula. As a result, this sample was influenced by the Iberian Peninsula’s PBL, in addition to the large amount of Saharan dust present at this time (Fig. 4B) which was previously transported by air masses at higher altitudes^33^.

The situation was completely different in the case of F28-HT (Fig. 4C). During this flight, samples were obtained at ~3,000 m, as in F24-HT but, as previously described, dust intrusion had finalized. Wind pattern above the Iberian Peninsula changed to predominant north-northwesterly winds, and precipitation favored dust settling on the Iberian Peninsula. Thus, back-trajectories computed via HYSPLIT showed predominant westerly air masses originating (72-hours) from the free troposphere above the Atlantic Ocean. A similar pattern was roughly observed in F28-LT (Fig. 4D), where the air masses also interacted with the Iberian Peninsula and marine PBL’s.

F9-HT back-trajectories showed predominant air masses originating from the Atlantic Ocean (72 h), after strong recirculation above the northwest side of the Iberian Peninsula (Fig. 4E), in addition to a direct interaction with lower atmospheric layers, including the PBL in some cases, due to the vertical movement of the air masses. This recirculation was also observed in F9-LT, although to a lesser extent, taking into account the transport of the air masses through the PBL (Fig. 4F). Back-trajectories for F10-LT and F10-HT are not shown here because they were similar to those presented for F9-LT and F9-HT, respectively. The summary of back-trajectory results as well as the predominant source for every sample is shown in Suplementary Table S1.

### Sequencing analysis and estimated richness

The collections of amplicons were sequenced, and the number of reads is summarized in Supplementary Table S2. After total DNA extraction from each of the collected samples, 16 S rDNA PCR amplification and DNA sequencing, a total of ∼1.5 ×10^6^ sequences were obtained across all samples, about 120,000 per sample on average. The analysis of the read length distributions indicated a modal distribution between 442 and 444 nt. After quality control checks, chimera sequence removal and controls sequences subtraction, a total of ∼140,000 sequences remained, about 11,000 per sample on average. Chloroplast sequences were detected (and removed) in all samples, with their proportion varying between 0.47 and 5.2%. The number of OTUs detected at a distance of 0.03, was between 30 to 375 per sample, with a total of 1,708 of bacterial OTUs observed. Nevertheless, Chao1estimations suggest that we were able to identify from 72 to 96% of the total OTU population. In general, samples taken during the flights showed lower number of OTUs than the surface samples (∼50% less).

Samples were collected at different altitudes (Surface, 100 m and 3,000 m) during three different atmospheric scenarios, a severe winter Saharan dust intrusion (23–24 February), a dust free environment after intense rains (28 February) and two weeks after the dust event (9–10 March). It is interesting to note that during dust event days, samples from the surface and flights showed a higher richness in bacterial population (Chao1) than the samples from non-dust event days. Conversely, although Shannon H diversity index showed higher values in the surface samples, similar values were found between dust-event days and non-dust samples. However, the Shannon H index was generally higher in surface samples than in the flight ones (∼47% less), except during the dust-event, in which no differences were found between surface and flight samples. Besides, sample F28-LT showed the lowest diversity (Shannon H index 0.5). Although similar studies of airborne bacterial diversity across different altitudes during a dust intrusion are currently nonexistent, our results are comparable to the diversity patterns observed in other studies dealing with desert dust events. Thus, samples collected during the dust intrusion showed a higher diversity (Chao1 and Shannon H index, Supplementary Table S2) than non-dust related samples, which is in agreement with other previous studies^4,13,30,34,35,60^. Additionally, rarefaction analysis (Suplementary Fig. S4) revealed that tropospheric communities are less complex compared to many habitats on Earth such as soils or waters^61^.

### Diversity of bacteria

**Figure 5:**
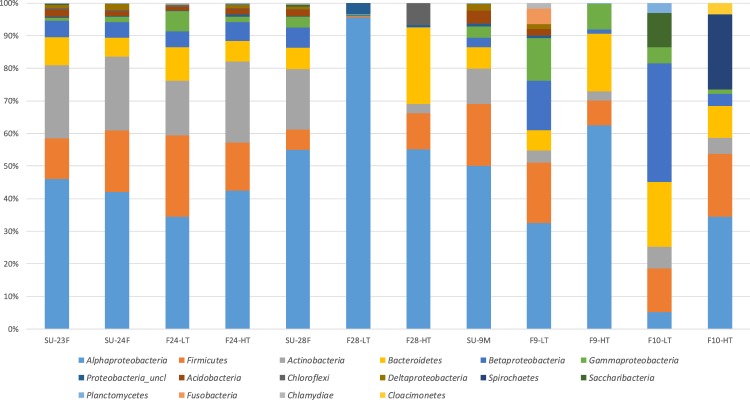
Relative abundance and Phylum-class level distribution of bacterial OTUs for each sampling. Only groups with relative abundances >1% are shown. Classification and histogram were performed by Mothur package.

Sequences were affiliated with 27 bacterial phyla and/or classes, among which *Alphaproteobacteria* (∼60%), *Actinobacteria* (∼10%), *Firmicutes* (∼10%) and *Bacteroidetes* (∼7%) were dominant, representing ∼90% of the total microbial community (Fig. 5, Suplementary Table S3). Approximately 7% of representative sequences could not be classified. Only six phyla were detected exclusively in the samples collected during the dust-event days, *Armatimonadetes*, Candidate division WPS- 1 and 2, *Nitrospirae*, *Parcubacteria* and *Synergistetes*, although each of them accounted for less than 1% of the sequences. In order to reveal any underlying trends in the composition of the different bacterial populations, hierarchical dendrogram at the phylum/class level (Suplementary Fig. S5) was generated via the Euclidean squared distance method. All the samples associated with dust intrusion (SU-23F, SU-24F, F24-HT and F24-LT) clustered together, and very near to the surface samples collected during the non-dust event (SU-28F and SU-9M). Additionally, samples from flights after the rain episode (F28-HT and F28-LT) grouped together, showing also the highest amounts of *Alphaproteobaterias* in F28-LT samples (∼90% of the total sequences) and *Chloroflexi* in F28-HT (∼9% of the total sequences). On the other hand, low troposphere flight samples from non-dust event day (F9-LT, F9-HT) as well as samples taken during the off-shore flights (F10-LT and F10-HT) showed a completely different microbial composition and clustered together. Samples from flight F9-LT revealed the highest amounts of *Fusobacteria* (96% of the total *Fusobacteria* related sequences) and *Cloacimonetes* were detected only in samples from off-shore flights (F10-LT and F10-HT). These samples also produced the highest percentages of *Spirochaetes* and *Saccharibacteria* (94% and 75% of the total respective phylum sequences).

**Figure 6:**
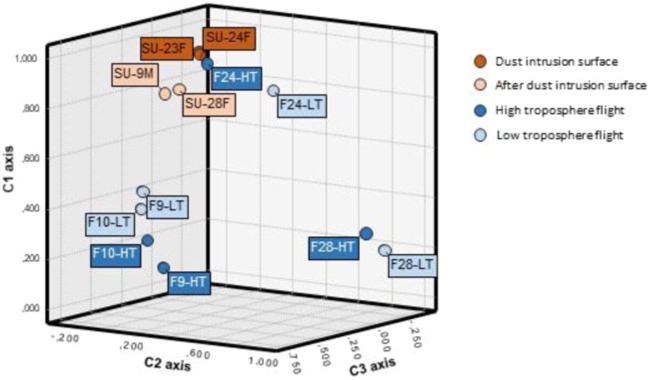
Principal Component Analyses (PCA) on the relative proportion of OTUs (at a 0.03 distance).

In turn, SSU rRNA gene sequences Principal Component Analysis (PCA) at 0.03 distance (Fig. 6), as well as the associated hierarchical clustering (Suplementary Fig. S6) was performed, explaining 71% of the total variance with three components. The PCA analysis revealed three major clusters among the samples, revealing that samples related in height or collection time seem to show more similar community composition patterns when compared to unrelated samples. The first cluster grouped all the surface samples (SU-23F, SU-24F, SU-28F and SU-9M) plus the samples from the first flight collected during the dust-event (F24-LT and F24-HT). This results reflected the influence of the dust intrusion in the microbial composition of the low troposphere, suggesting that desert dust intrusions aerosolize a large number of new cells and taxa, dramatically affecting the composition of the low tropospheric microbial communities, at least up to the 3,000 m sampled. Back trajectory analysis (22–23 Feb) showed that air mases coming from North Africa (and microbial cells transported with them) experienced a strong vertical mixing with different atmospheric layers, including the Saharan Planetary boundary layer (PBL) in origin, and were brought aloft during the dust intrusion event. This could explain the similarity among the samples taken at different altitudes during the intrusion.

Interestingly, surface samples from days after the dust intrusion event (SU-28F and SU-9M) are also included in this cluster (although forming a subcluster), showing a clear similarity with the microbial community during the dust intrusion (Fig. 6). This result suggest that, changes in the local atmospheric microbial composition near the surface caused by the dust intrusions may remain for several days after the major event has passed^60^. This desert influence in the atmosphere could extend even to several weeks in the presence of thermal inversion phenomena, which are frequently associated with hot air desert intrusions in the Iberian Peninsula during the winter^62^.

The Saharan origin of these microbial communities is revealed by the high conformity of principal bacterial genera described for Saharan desert with those present in these samples^63^. Thus, the most abundant bacterial species in all samples taken during the dust event (∼30–40% of the total sequences in these samples) (Suplementary Table S3), gruped in three different phyla, previously reported during desert dust events^35,60,63–65^: (a) *Proteobacteria*-*Rhizobiales* (*Rhizobium* and *Microvirga*), *Sphingomonadales*, *Rhodobacterales* (*Rubellimicrobium*), (b) *Actinobacteria*- *Geodermatophilaceae*; (c) *Firmicutes*- *Bacillaceae*,. Most of these species are well known for being extremely stress-resistant, including, high temperatures, such as *Rubellimicrobium*^63^, high radiation and desiccation, specially *Geodermatophilus*^64,65^, or spore formation such as *Bacillus*, which increase the probability of survival during transportation^66^.

Additionally, other highly stress-resistant bacteria groups were also detected, although in low amounts, representing always less than 1% of the total sequences in each sample. Thus, species related to, (a) high temperature: *Pseudonocardia*, *Streptomyces*, *Thermomonosporaceae*, *Nitrospira* and *Porphyrobacter*; (b) radiation: *Deinococcus*, *Hymenobacter*, *Methylobacterium* and *Rubrobacter*; (c) desiccation: *Kineococcus* and *Methylobacterium*; (d) psychrotolerance: *Planococcus*, *Subtercola* and *Modestobacter*; and (e) halophyly: *Salinarimonas* and *Nocardioides*, were also detected in all the samples related to the dust event (SU-23F, SU-24F, F24-LT, F24-HT) as well as in the surface samples taken after it (SU-28F, SU-9M).

The second cluster observed in the PCA analysis (Fig. 6 and Suplementary Fig. S6) grouped the samples obtained during the second flight, after the rain event (F28-LH and F28-HT), pointing out a completely different situation. After the rain, the atmospheric microbial community from samples collected during the flights changed completely, with samples being clearly related by time scale rather than altitude. Thus, samples from flights after the rain period (F28-LT and F28-HT) clustered together and separately from the samples taken during the flights two weeks after the dust event (F9-LT, F9-HT, F10-LT and F10-HT). These results reflected the completely different atmospheric situation once the dust intrusion was over. Winds pattern above the Iberian Peninsula changed to predominant north-northwesterly winds, and the precipitations favored dust deposition. Our results are in agreement with previous studies in which it has been hypothesized that an important variable affecting the differences in atmospheric bacterial composition may be due to variability in the predominant winds at any given time^64,65^. In these samples, *Rhizobium* is the main represented genera (∼40–90% of the total sequences in these samples). *Rhizobium* is a sporulating Gram-negative soil bacteria that form endosymbiotic nitrogen-fixing associations with plant roots such as legumes^67^. Consistent analysis from previous studies reported that, in continental and temperate regions, plants are one of the main sources of atmospheric bacteria. This is suggested by the large quantity of plant associated bacteria, such as *Sphingomonadales* and *Rhizobiales*, and the high number of chloroplast sequences retrieved in the samples collected (data not shown). Besides, a previous study demonstrated that soil-inhabiting bacteria, such as *Actinobacteriales*, *Firmicutes* and *Rhizobiales*, prevailed in the atmosphere during cold seasons^68^. In our case, it is tentative to suggest that the abundance of *Rhizobiales* in the atmosphere could be a result of the land area surrounding the flight samplings, which are mainly used for agricultural purposes, where seasonal plowing and cultivation processes may contribute to the atmospheric bacterial sources^69^. *Rhizobiales* were also found in the dust event related samples, but represented less than the 5% of the total sequences found.

PCA analysis also revealed that the microbial communities in the samples taken during the flights that took place 15 days after the dust event (F9-HT, F9-LT, F10-HT and F10-LT) were much more similar among them when compared to the other samples collected. In this case, *Brevundimonas*, and *Methylobacterium* as well as *Cupriavidus* and *Mesorizobium* were the most abundant genera respectively (∼20–50% of the total sequences in these samples) (Fig. 6 and Suplementary Fig. S6). *Brevundimonas* species are ubiquitous in the environment and is one of few bacteria showing high survival rates under radiation simulating Martian conditions, while some species of *Cupriavidus* are known by their high resistance to oxidative stress^70,71^.

Our results showed that, despite the similitudes in the bacterial diversity between our atmospheric samples and the previously reported ones, there are a number of important and stark differences. For example, we did not find any sequence related to *Afipia* sp., one of the main constituents of the troposphere community (>40% of total community) reported by DeLeon-Rodriguez *et al*. (2013), as well as some of the most abundant families recently described by Smith *et al*. (2018) in the lower stratosphere (such as *Staphylococcaceae*, *Moraxellaceae*, *Lachnospiraceae* and *Ruminococcaceae*), although we found a similar number of OTUS. Furthermore, one of the main components found in our samples related to Saharan intrusion, *Microvirga* sp., has not been reported previously in the consulted literature. Additionally, contrary to previous studies, we did not find what has been described as a “core microbiome”^36^ since there are no bacterial species common to all the samples analyzed, not even within the flight samples.

On the other hand, Triado-Margarit *et al*. (2019) reported that the bacterial composition of bioaerosols collected by passive natural deposition at high-elevated mountains were closer to the bacterial microbiome from the free troposphere. Our results showed also comparable patterns, since samples from the same flights at low and high troposphere were similar in terms of bacterial diversity. Besides, our analysis showed a different bacterial community structure in samples collected over the open ocean, during desert dust events or after a rain period, as described in the mentioned study^72^. Interestingly, these authors stated that only *Oxalobacteraceae* were notably more abundant in wet deposition, with *Noviherbaspirillum* and *Massilia* as dominant genera^72^. In our case, although these genera have been found in most of the samples, they account for less than 1% of the total community in all cases. These examples support the idea of a highly dynamic and diverse atmospheric microbiome that deserve considerably more attention due to the different bioaerosol origins, the assortment of known emission sources and the potential for long range atmospheric dispersal^31^.

### Potential health threats

After long-range transport, the diversity of bacteria associated with dust events may play a significant role in human, plant and animal health, although it is not clear what are the limits on the overall transport of pathogens around the planet. We have found that most of the potential pathohogenic-relevant bacteria are mainly found in the dust event related samples, in both surface and flight samples. Although HtS methods did not permit assigning species, several environmental genera that contain opportunistic pathogens^25,27,73^, were identified including *Legionella*, *Neisseria*, *Staphylococcus*, *Chlamydia*, *Acinetobacter*, *Arthrobacter*, *Gordonia*, *Kocuria*, *Brevibacterium*, *Rasltonia*, *Pantoea*, *Pseudomonas*, *Mycobacterium* and *Bacillus*.

Special attention should be paid to the samples taken during the flights carried out on 9 March, in which approximately 50% of the sequences corresponded to the genus *Brevundimonas*, a Gram-negative bacterium recently described as an emerging global opportunistic pathogen in nosocomial infections^72^. Furthermore, sequences related to *Brucella* (associated to brucellosis) and *Haemophilus* (associated to bacterial flu) genera were also found in significant amounts in these flight samples. Although no reports of human infectious diseases related to long-distance dispersal of pathogens associated to dust events exists to date, aside from increasing incidences of asthma, the World Health Organization has reported dust storm events in the sub-Sahara region as one of the main origins for local occurrences of meningococcal meningitis (*Neisseria meningitidis*), causing around 50,000 deaths every year^73^. Thus, enhanced desertification will introduce more dust into the atmosphere, increasing the importance of monitoring microbial communities in intercontinental winds. This is especially relevant in terms of the aerial dispersal of plant pathogens, due to the limited genetic diversity of the majority of the modern crops that make them highly susceptible to new pathogens^17,20^.

Overall, we observed that samples related in height or time scale showed more similar community composition patterns compared with unrelated samples. Additionally, the dust intrusion had a profound influence in the microbial composition of free trophosphere areas with little influence of ground surface contamination, such as areas located up to 3,000 m. In this study, Sahara desert storms were able to aerosolize a large number of cells and species that have been distributed along the entire air column, strongly resembled the bioaerosols collected near the ground even 15 days after the dust event. There is still a lack of knowledge about the atmospheric microbial community, its spatial and temporal distribution, how they can adapt to this environment and wheter they are able to actively metabolize organic and inorganic compounds and proliferate in order to consider the atmosphere as an ecosystem in its own right rather than as a conduit for life. It is also important to understand how atmospheric phenomena (i.e. dust storms, hurricanes, etc…) suspend and transport microbial communities globally. For this reason, there is a need for improvement and implementation of suitable sampling systems and the development of manned and unmanned aerial systems capable of sampling large volumes of air for subsequent analyses, as it would provide a wide range of applications in atmospheric, environmental and health sciences.

## Supplementary information

Supplementary Information.

Supplementary Table S3

## References

[1] Smets W, Moretti S, Denys S, Lebeer S (2016). Airborne bacteria in the atmosphere: presence, purpose, and potential. Atmos. Environ..

[2] Fröhlich-Nowoisky J (2016). Bioaerosols in the Earth system: Climate, health, and ecosystem interactions. Atmos. Res..

[3] Morris CE (2014). Bioprecipitation: A feedback cycle linking Earth history, ecosystem dynamics and land use through biological ice nucleators in the atmosphere. Glob. Chang. Biol..

[4] Polymenakou PN, Mandalakis M, Stephanou EG, Tselepides A (2008). Particle size distribution of airborne microorganisms and pathogens during an intense African dust event in the Eastern Mediterranean. Environ. Health Perspect..

[5] Pillai SD, Ricke SC (2002). Bioaerosols from municipal and animal wastes: background and contemporary issues. Can. J. Microbiol..

[6] Hervàs A, Camarero L, Reche I, Casamayor EO (2009). Viability and potential for immigration of airborne bacteria from Africa that reach high mountain lakes in Europe. Environ. Microbiol..

[7] Moorman JE (2011). Current asthma prevalence in United States, 2006-2008. MMWR Surveill Summ.

[8] Lighthart B (2000). Mini-review of the concentration variations found in the alfresco atmospheric bacterial populations. Aerobiologia.

[9] Gonzalez-Martin C, Teigell-Perez N, Lyles M, Valladares B, Griffin DW (2013). Epifluorescent direct counts of bacteria and viruses from topsoil of various desert dust storm regions. Res. Microbiol..

[10] Cáliz J, Triadó-Margarit X, Camarero L, Casamayor EO (2018). A long-term survey unveils strong seasonal patterns in the airborne microbiome coupled to general and regional atmospheric circulations. Proc. Natl. Acad. Sci..

[11] Smith DJ (2013). Microbes in the upper atmosphere and unique opportunities for astrobiology research. Astrobiology.

[12] Smith DJ, Griffin DW, Schuerger AC (2010). Stratospheric microbiology at 20 km over the Pacific Ocean. Aerobiologia.

[13] Griffin DW (2007). Atmospheric movement of microorganisms in clouds of desert dust and implications for human health. Clin. Microbiol. Rev..

[14] Reche I, D’Orta G, Mladenov N, Winget DM, Suttle CA (2018). Deposition rates of viruses and bacteria above the atmospheric boundary layer. ISME J..

[15] Okin, G. S., Mahowald, N., Chadwick, O. A. & Artaxo, P. Impact of desert dust on the biogeochemistry of phosphorus in terrestrial ecosystems. *Global Biogeochem. Cycles* **18** (2004).

[16] Duarte, C. M. *et al*. Aerosol inputs enhance new production in the subtropical northeast Atlantic. *J. Geophys. Res. Biogeosciences* **111** (2006).

[17] Kellogg CA, Griffin DW (2006). Aerobiology and the global transport of desert dust. Trends Ecol. Evol..

[18] Moulin C, Lambert CE, Dulac F, Dayan U (1997). Control of atmospheric export of dust from North Africa by the North Atlantic oscillation. Nature.

[19] Shinn EA (2000). African dust and the demise of Caribbean coral reefs. Geophys. Res. Lett..

[20] Brown JKM, Hovmøller MS (2002). Aerial dispersal of pathogens on the global and continental scales and its impact on plant disease. Science.

[21] Prospero JM, Blades E, Mathison G, Naidu R (2005). Interhemispheric transport of viable fungi and bacteria from Africa to the Caribbean with soil dust. Aerobiologia.

[22] Foltz GR, McPhaden MJ (2008). Impact of Saharan dust on tropical North Atlantic. J. Clim..

[23] Bangert M (2012). Saharan dust event impacts on cloud formation and radiation over Western Europe. Atmos. Chem. Phys..

[24] d’Almeida GA (1986). A Model for Saharan dust transport. J. Clim. Appl. Meteorol..

[25] Dulac, F. * et al.* Quantitative remote sensing of African dust transport to the Mediterranean in The impact of desert dust across the Mediterranean (eds. Guerzoni, S. & Chester, R.) 25–49 (Springer Netherlands, 1996).

[26] Alpert P, Kishcha P, Shtivelman A, Krichak S, Joseph J (2004). Vertical distribution of Saharan dust based on 2.5-year model predictions. Atmos. Res..

[27] Griffin DW, Garrison VH, Herman JR, Shinn EA (2001). African desert dust in the Caribbean atmosphere: Microbiology and public health. Aerobiologia.

[28] Weir-Brush JR, Garrison VH, Smith GW, Shinn EA (2004). The relationship between Gorgonian coral (Cnidaria: Gorgonacea) diseases and African dust storms. Aerobiologia.

[29] Griffin, D. W., Gonzalez-Martin, C., Hoose, C. & Smith, D. J. Global scale atmospheric dispersion of microorganisms in Microbiology of Aerosols (eds. Delort, A. & Amato, P.) 155–194 (John Wiley & Sons, Inc., 2017).

[30] Weil T (2017). Legal immigrants: invasion of alien microbial communities during winter occurring desert dust storms. Microbiome.

[31] Smith DJ (2018). Airborne bacteria in Earth’s lower stratosphere resemble taxa detected in the Troposphere: results from a new NASA aircraft bioaerosol collector (ABC). Front. Microbiol..

[32] Yamaguchi N, Ichijo T, Sakotani A, Baba T, Nasu M (2012). Global dispersion of bacterial cells on Asian dust. Sci. Rep..

[33] Kaskaoutis DG, Nastos PT, Kosmopoulos PG, Kambezidis HD (2012). Characterising the long-range transport mechanisms of different aerosol types over Athens, Greece during 2000–2005. Int. J. Climatol..

[34] Rosselli R (2015). Microbial immigration across the Mediterranean via airborne dust. Sci. Rep..

[35] Sánchez de la Campa A, García-Salamanca A, Solano J, de la Rosa J, Ramos JL (2013). Chemical and microbiological characterization of atmospheric particulate matter during an intense African dust event in Southern Spain. Environ. Sci. Technol..

[36] DeLeon-Rodriguez N (2013). Microbiome of the upper troposphere: Species composition and prevalence, effects of tropical storms, and atmospheric implications. Proc. Natl. Acad. Sci..

[37] Kobayashi F (2015). Bioprocess of Kosa bioaerosols: Effect of ultraviolet radiation on airborne bacteria within Kosa (Asian dust). J. Biosci. Bioeng..

[38] Salter SJ (2014). Reagent and laboratory contamination can critically impact sequence-based microbiome analyses. BMC Biol..

[39] Minich JJ (2018). KatharoSeq enables High-Throughput microbiome analysis from low-biomass samples. mSystems.

[40] Stein AF (2015). NOAA’s HYSPLIT atmospheric transport and dispersion modeling system. Bull. Am. Meteorol. Soc..

[41] Rolph G, Stein A, Stunder B (2017). Real-time environmental applications and display system: READY. Environ. Model. Softw..

[42] Klindworth A (2013). Evaluation of general 16S ribosomal RNA gene PCR primers for classical and next‐generation sequencing‐based diversity studies. Nucleic Acids Res.

[43] Schloss PD (2009). Introducing mothur: open-Source, platform-independent, community-supported software for describing and comparing microbial communities. Appl. Environ. Microbiol..

[44] Kozich JJ, Westcott SL, Baxter NT, Highlander SK, Schloss PD (2013). Development of a dual-Index sequencing strategy and curation pipeline for analyzing amplicon sequence data on the MiSeq Illumina sequencing platform. Appl. Environ. Microbiol..

[45] Pruesse E (2007). SILVA: a comprehensive online resource for quality checked and aligned ribosomal RNA sequence data compatible with ARB. Nucleic Acids Res..

[46] Altschul SF, Gish W, Miller W, Myers EW, Lipman DJ (1990). Basic local alignment search tool. J. Mol. Biol..

[47] Cole JR (2008). The Ribosomal Database Project: improved alignments and new tools for rRNA analysis. Nucleic Acids Res..

[48] Hammer, O., Harper, D. A. T. & Ryan, P. D. Palaeontological statistics software package for education and data analysis. Palaeontol. Electron. 4, (2001).

[49] Braak, C. J. F. & Šmilauer, P. CANOCO reference manual and CanoDraw for Windows user’s guide. Softw. canonical community ordination (version 4.5). Microcomput. Power, Ithaca, NY 500 (2002).

[50] Goudie AS (2009). Dust storms: recent developments. J. Environ. Manage..

[51] Swap R, Garstang M, Greco S, Talbot R, Kallberg P (1992). Saharan dust in the Amazon Basin. Tellus B.

[52] Gallisai R, Peters F, Volpe G, Basart S, Baldasano JM (2014). Saharan dust deposition may affect phytoplankton growth in the Mediterranean Sea at ecological time scales. PLoS One.

[53] Barberán A, Henley J, Fierer N, Casamayor EO (2014). Structure, inter-annual recurrence, and global-scale connectivity of airborne microbial communities. Sci. Total Environ..

[54] Griffin DW (2007). Airborne desert dust and aeromicrobiology over the Turkish Mediterranean coastline. Atmos. Environ..

[55] Gat D, Mazar Y, Cytryn E, Rudich Y (2017). Origin dependent variations in the atmospheric microbiome community in Eastern Mediterranean dust storms. Environ. Sci. Technol..

[56] Moreno T (2006). Geochemical variations in aeolian mineral particles from the Sahara–Sahel dust corridor. Chemosphere.

[57] Alastuey A (2005). Characterisation of TSP and PM2.5 at Izaña and Sta. Cruz de Tenerife (Canary Islands, Spain) during a Saharan Dust Episode (July 2002). Atmos. Environ..

[58] Rodríguez S (2011). Transport of desert dust mixed with North African industrial pollutants in the subtropical Saharan Air Layer. Atmos. Chem. Phys..

[59] Kallos G, Astitha M, Katsafados P, Spyrou C (2007). Long-Range transport of anthropogenically and naturally produced particulate matter in the Mediterranean and North Atlantic: current state of knowledge. J. Appl. Meteorol. Climatol..

[60] An S, Sin HH, DuBow MS (2014). Modification of atmospheric sand-associated bacterial communities during Asian sandstorms in China and South Korea. Heredity (Edinb)..

[61] Delmont TO, Simonet P, Vogel TM (2012). Describing microbial communities and performing global comparisons in the ‘omic era. Isme J..

[62] Queroll Carceller X, Urós AA, Fernández SC (2008). Impacto de las emisiones desérticas de polvo africano sobre la calidad del aire en españa. Macla Rev. la Soc. Española Mineral..

[63] Favet J (2012). Microbial hitchhikers on intercontinental dust: catching a lift in Chad. Isme J..

[64] Cao C (2014). Inhalable microorganisms in Beijing’s PM2.5 and PM10 pollutants during a severe smog event. Environ. Sci. Technol..

[65] Rainey FA (2005). Extensive diversity of ionizing-radiation-resistant bacteria recovered from Sonoran Desert soil and description of nine new species of the genus Deinococcus obtained from a single soil sample. Appl. Environ. Microbiol..

[66] Nicholson WL, Munakata N, Horneck G, Melosh HJ, Setlow P (2000). Resistance of Bacillus endospores to extreme terrestrial and extraterrestrial environments. Microbiol. Mol. Biol. Rev..

[67] Sawada H, Kuykendall LD, Young JM (2003). Changing concepts in the systematics of bacterial nitrogen-fixing legume symbionts. J. Gen. Appl. Microbiol..

[68] Gandolfi I, Bertolini V, Ambrosini R, Bestetti G, Franzetti A (2013). Unravelling the bacterial diversity in the atmosphere. Appl. Microbiol. Biotechnol..

[69] Bowers RM (2013). Seasonal variability in bacterial and fungal diversity of the near-surface atmosphere. Environ. Sci. Technol..

[70] Nies DH (1999). Microbial heavy-metal resistance. Appl. Microbiol. Biotechnol..

[71] Dartnell LR, Hunter SJ, Lovell KV, Coates AJ, Ward JM (2010). Low-Temperature ionizing radiation resistance of Deinococcus radiodurans and Antarctic Dry Valley bacteria. Astrobiology.

[72] Triadó-Margarit X, Caliz J, Reche I, Casamayor EO (2019). High similarity in bacterial bioaerosol compositions between the free troposphere and atmospheric depositions collected at high-elevation mountains. Atmospheric Environment..

[73] Griffin DW, Kellogg CA, Shinn EA (2001). Dust in the wind: long range transport of dust in the atmosphere and its implications for global public and ecosystem health. Glob. Chang. Hum. Heal..

