# Supplementary material for: Impacts of Saharan Dust Intrusions on Bacterial Communities of the Low Troposphere
Source: Sci Rep. 2020 Apr 22;10:6837. doi: 10.1038/s41598-020-63797-9 (PMC7176723; doi:10.1038/s41598-020-63797-9)
Supplement: Supplementary file 1 — Supplementary Information. [file 41598_2020_63797_MOESM1_ESM.docx]

**SUPPORTING INFORMATION**

**Impacts of Saharan Dust Intrusions on Bacterial Communities of the Low Troposphere**

Elena González-Toril^a^, Susana Osuna^a^, Daniel Viúdez-Moreiras^a^, Ivan Navarro-Cid^a^, Silvia Díaz del Toro^b^, Sutyvann Sor Mendi^c^, Rafael Bardera^c^, Fernando Puente-Sánchez^d^, Graciela de Diego-Castilla^a^, Ángeles Aguilera^a^*

^a^ Centro de Astrobiología (CSIC-INTA). Carretera de Ajalvir Km 4, Torrejón de Ardoz. 28850 Madrid, Spain.

^b^ Department of Genetics, Physiology and Microbiology. Biology Faculty. C/José Antonio Novais, 12, Universidad Complutense de Madrid (UCM), 28040, Madrid, Spain.

^c^ Aerodinamic Department (INTA). Carretera de Ajalvir Km 4, Torrejón de Ardoz. 28850 Madrid, Spain.

^d^ Systems Biology Program. Centro Nacional de Biotecnología. C/ Darwin nº 3, Campus de Cantoblanco 28049 Madrid, España.

^*^**Corresponding author**: Ángeles Aguilera. Mailing address: Centro de Astrobiología (CSIC-INTA). Carretera de Ajalvir Km 4, Torrejón de Ardoz. 28850 Madrid, Spain. Email: aguileraba@cab.inta-csic.es Phone: (34)91 520 6434; Fax: (34)91 520 1034

**Table S1.-** Back-trajectory results and predominant sources for each flight.

| **Flight Code** | **Date** | **Predominant air mass source (3-days backward trajectories)** | **Link to the predominant sample source** |
| --- | --- | --- | --- |
| F24-HT | 24/02/2017 | North Africa, after strong vertical mixing within the Saharan's PBL* (Feb 22 - Feb 23) Atlantic Ocean (Feb 24) | Saharan's PBL + contribution from Atlantic Ocean |
| F24-LT | 24/02/2017 | PBL from the east side of the Iberian Peninsula and Spain's Mediterranean coast (Feb 22 - Feb 23) PBL from the northwest side of the Iberian Peninsula (Feb 24) | PBL from the Iberian Peninsula and Spain's Mediterranean coast + Saharan's PBL dust |
| F28-HT | 28/02/2017 | Atlantic Ocean | Atlantic Ocean |
| F28-LT | 28/02/2017 | Atlantic Ocean | Atlantic Ocean + possibly some residual Saharan's PBL dust + PBL from the Iberian Peninsula |
| F9-HT | 09/03/2017 | Atlantic Ocean, after strong recirculation above the northwest side of the Iberian Peninsula | Atlantic Ocean and PBL from the northwest side of the Iberian Peninsula |
| F9-LT | 09/03/2017 | Atlantic Ocean, after weak recirculation in the northwest side of the Iberian Peninsula | Atlantic Ocean and PBL from the northwest side of the Iberian Peninsula |
| F10-HT | 10/03/2017 | Atlantic Ocean, after strong recirculation above the west side of the Iberian Peninsula | Atlantic Ocean + possibly PBL from the west side of the Iberian Peninsula |
| F10-LT | 10/03/2017 | Atlantic Ocean, after weak recirculation in the north-northwest side of the Iberian Peninsula | Atlantic Ocean and PBL from the northwest side of the Iberian Peninsula |

* Planetary Boundary Layer

| **Sample*** | **Initial number of sequences** | **% of removed chimeras** | **Sequences final number **** | **OTUs ***** | **Chao-1** | **Shannon-H Index** |
| --- | --- | --- | --- | --- | --- | --- |
| SU-23F | 164,328 | 3.4 | 14,339 | 375 | 464.1 | 4.3 |
| SU-24F | 176,508 | 3.1 | 13,010 | 340 | 427.3 | 4.3 |
| F24-HT | 152,630 | 1.5 | 11,423 | 225 | 236 | 4.1 |
| F24-LT | 166,191 | 0.8 | 11,806 | 184 | 205 | 4.4 |
| SU-28F | 166,542 | 1.6 | 6,729 | 199 | 211.4 | 3.9 |
| F28-HT | 153,500 | 4.1 | 11,845 | 33 | 38 | 2.2 |
| F28-LT | 184,770 | 0.5 | 44,021 | 76 | 105 | 0.5 |
| SU-9M | 194,843 | 1.3 | 8,104 | 98 | 102 | 3.6 |
| F9-HT | 142,752 | 0.4 | 5,340 | 30 | 33.75 | 1.9 |
| F9-LT | 139,245 | 0.9 | 3,914 | 79 | 84.6 | 3.4 |
| F10-HT | 140,481 | 0.6 | 3,840 | 31 | 32.67 | 2.4 |
| F10-LT | 144,111 | 0.6 | 3,362 | 38 | 48.5 | 2.7 |

**Table S2.** Sequencing data. Total numbers of reads accumulated for each sample in the original datasets after filtering and classification. Number of total bacterial OTUs and diversity indices.

* Sample: (SU) Surface; (F) Flight; (HT) High Troposphere flight; (LT) Low Troposphere flight

** Number of sequences after quality-filtering steps, chimeras, controls and chloroplast removal

*** OTUs number at 0.03 level


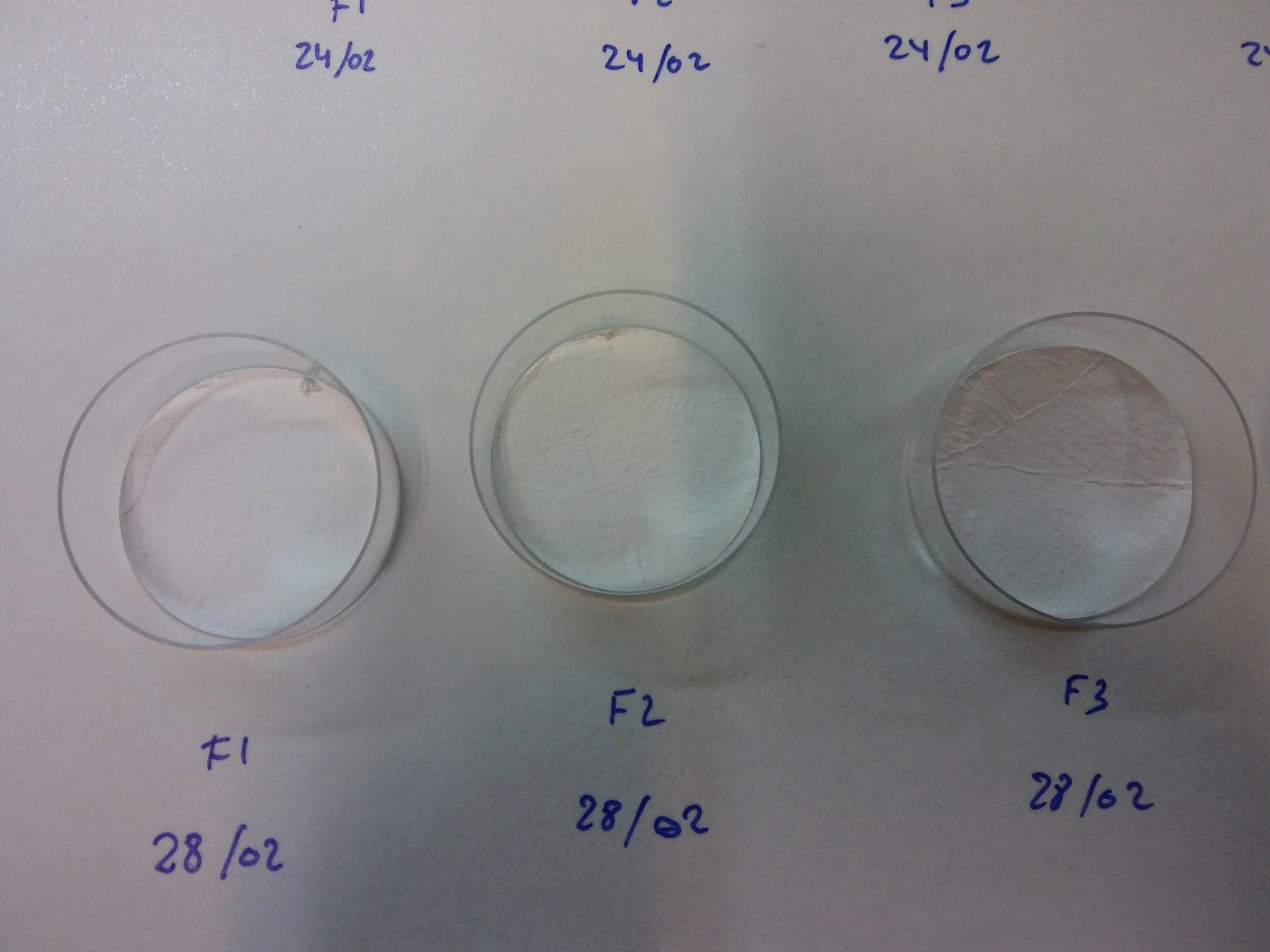


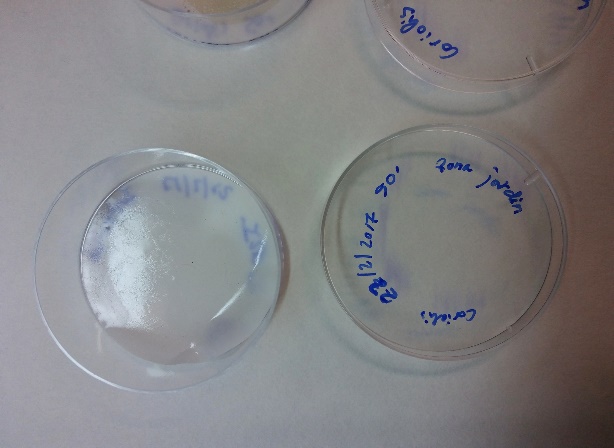

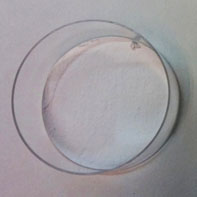

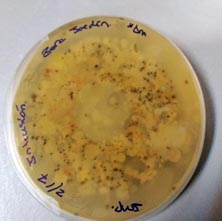

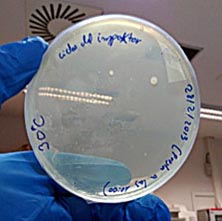

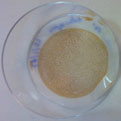

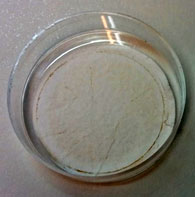


**A**

**B**

**C**

**D**

**E**

**F**

28 de febrero

**Figure S1**. Photographs of the filters after sampling. (A) General vision of the filter after F24-LT (100 m) flight; (B) Filter after F24-HT (3,000 m) flight; (C) Filter after F28-LT (100 m) flight; (D) Filter after F28-HT (3,000 m) flight; (E) Cultivation plate from F24-LT sample after 24 hr at 30ºC showing more than 100 colonies; (F) Cultivation plate from F28-LT sample after 24 hr at 30ºC showing less than 10 colonies.


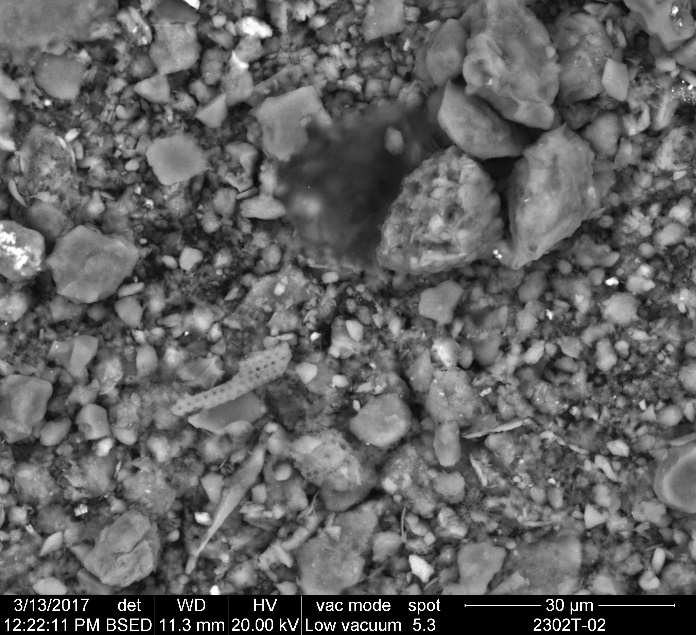


**30 μm**

**A**


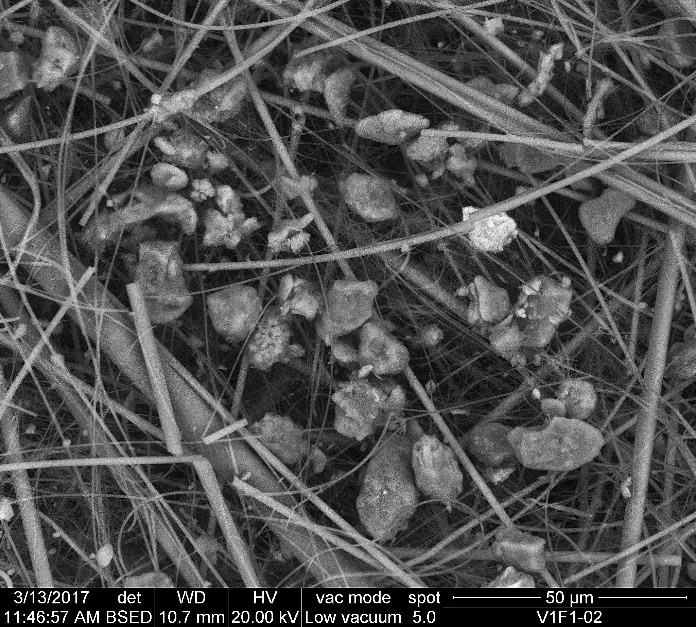


**50 μm**

**B**

**E**

**F**

**D**

**C**


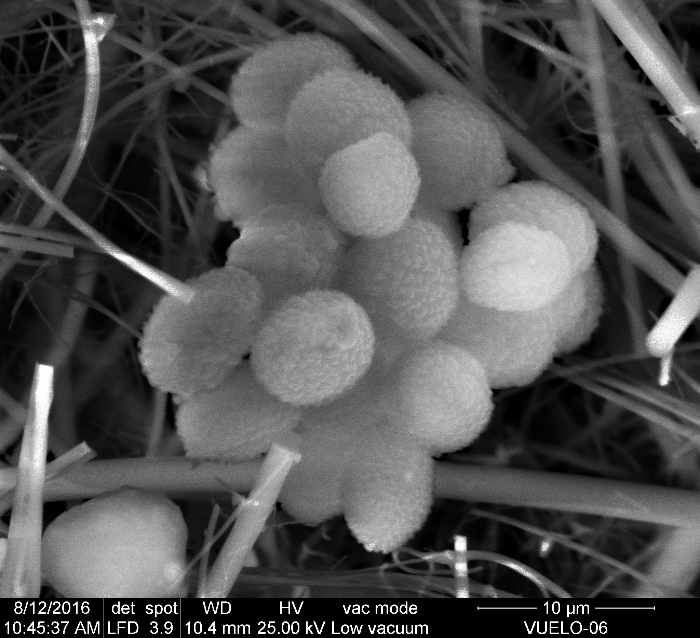


**10 μm**


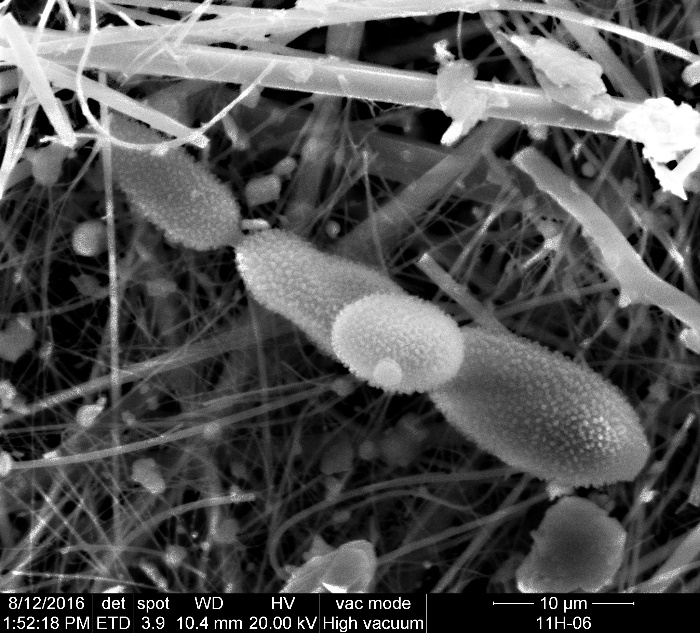


**10 μm**


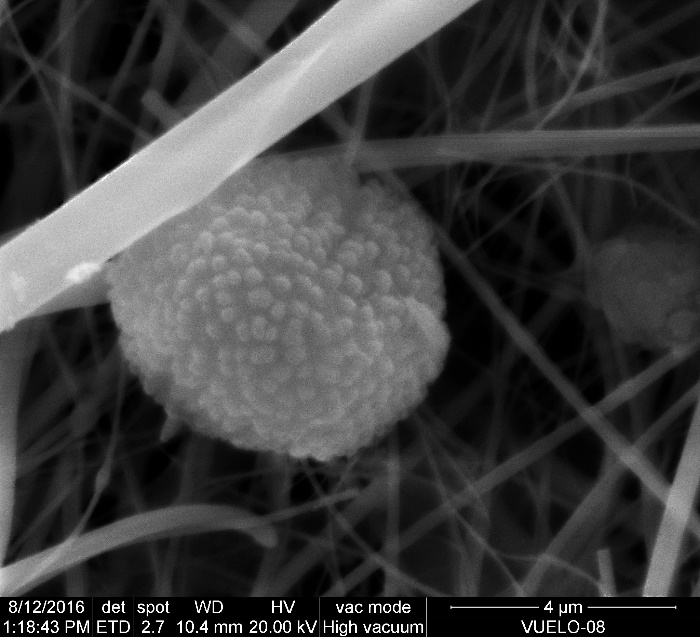


**4 μm μm**


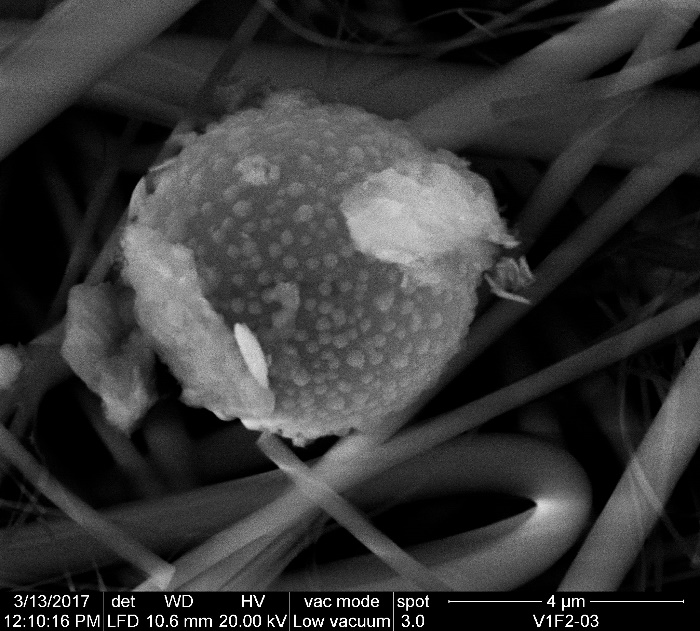


**4 μm**

**10 μm**

**Figure S2.** SEM micrographs of air samples. (A) general vision of the filter SU-24F after sampling during the dust intrusion, showing the morphology and grain size of particles samples; (B) general vision of the filter after the flight sampling F24-HT at 3,000 m, showing lower but significant amounts of particulate grains; (C, D) coccoid microbial cells surrounded by a dense coat of Extracellular Polymeric Substances (EPSs); (E, F) microbial colonies of different cells found during the flight F24-LT at 100 m over the surface. Again, the coat of EPSs is also visible.


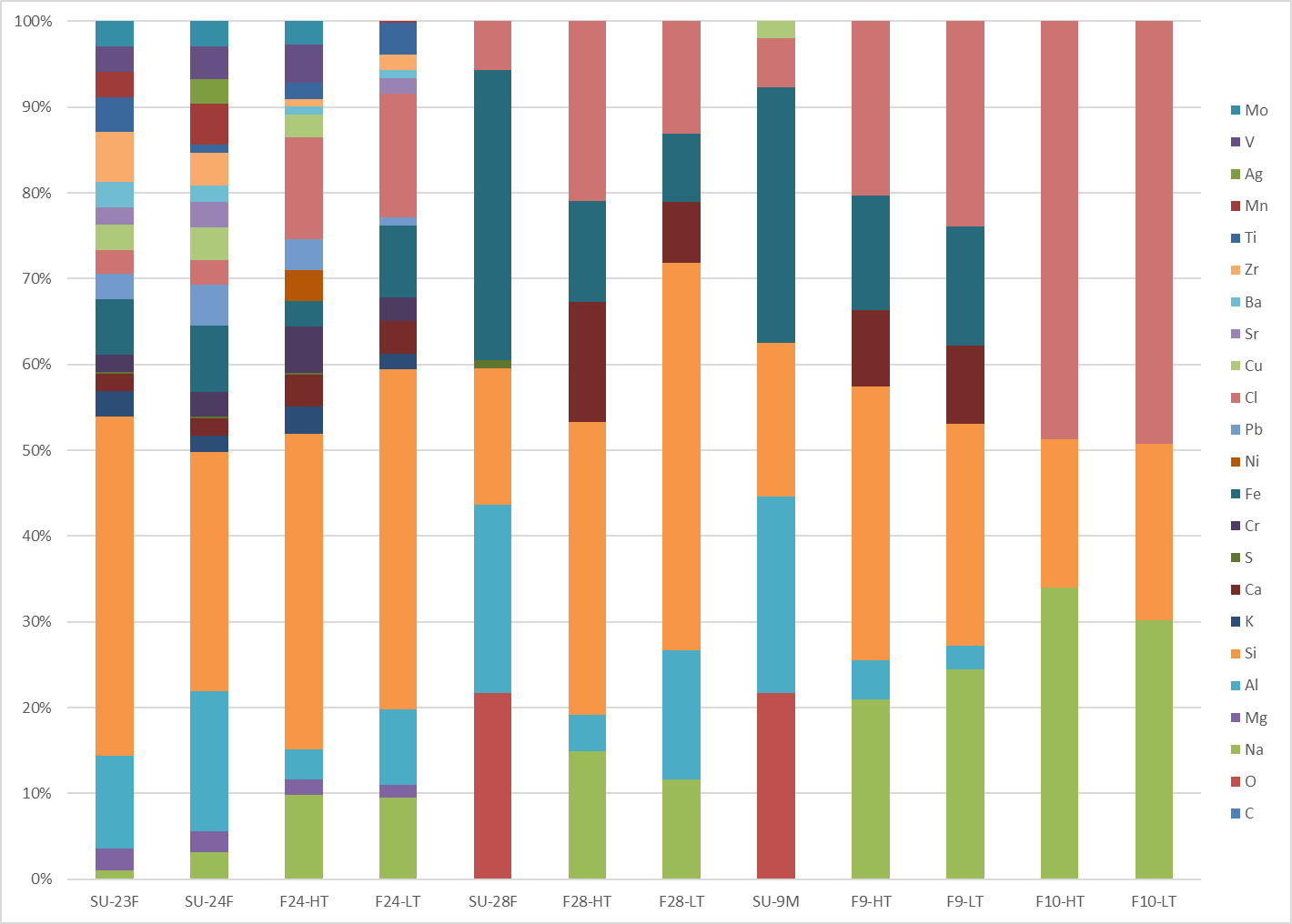


**Figure S3.** EDX analysis. Percentages of chemical element weights (wt%) which corresponds to the absolute concentration of each element in every sample.


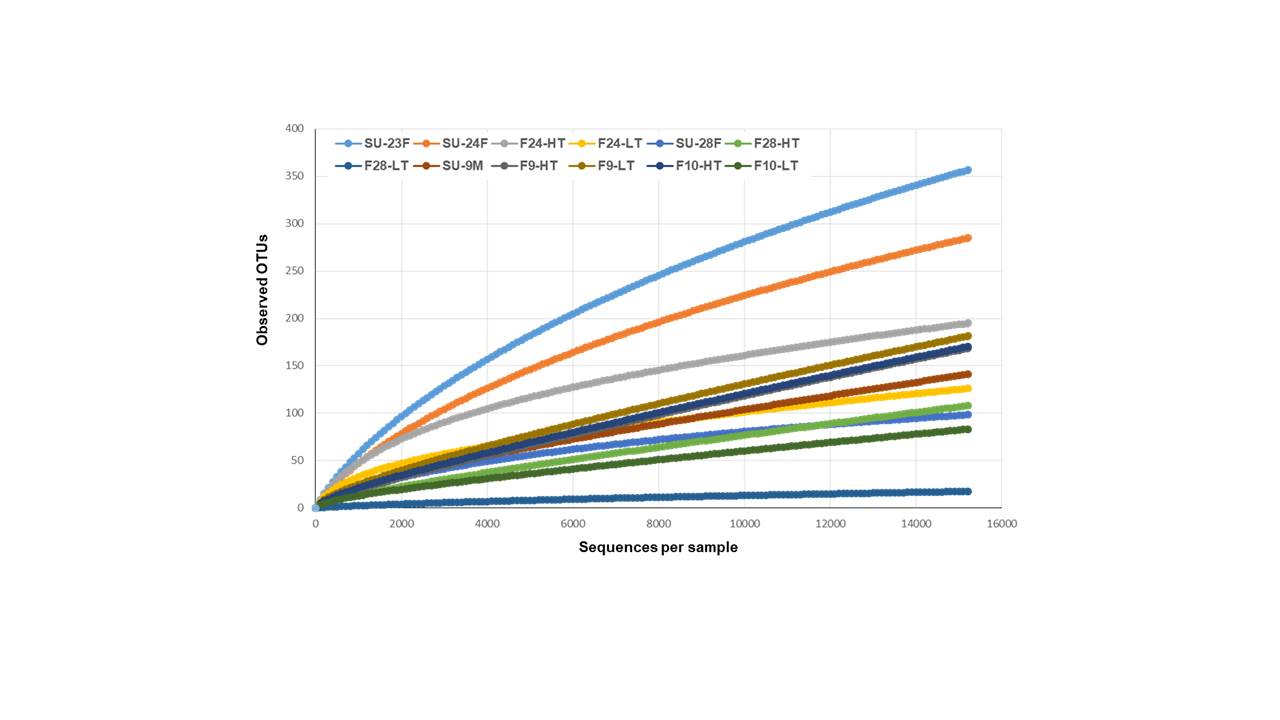


**Figure S4.** Rarefraction curves of SSU rRNA gene amplicon sequences recovered from the samples. Curves represent the number of unique OTUs recovered (vertical axis), defined at the 97% nucleotide sequence identity level, for the number of sequences analyzed (horizontal axis) and reflect the extent of OTU diversity within the samples and what fraction of this diversity was sampled.


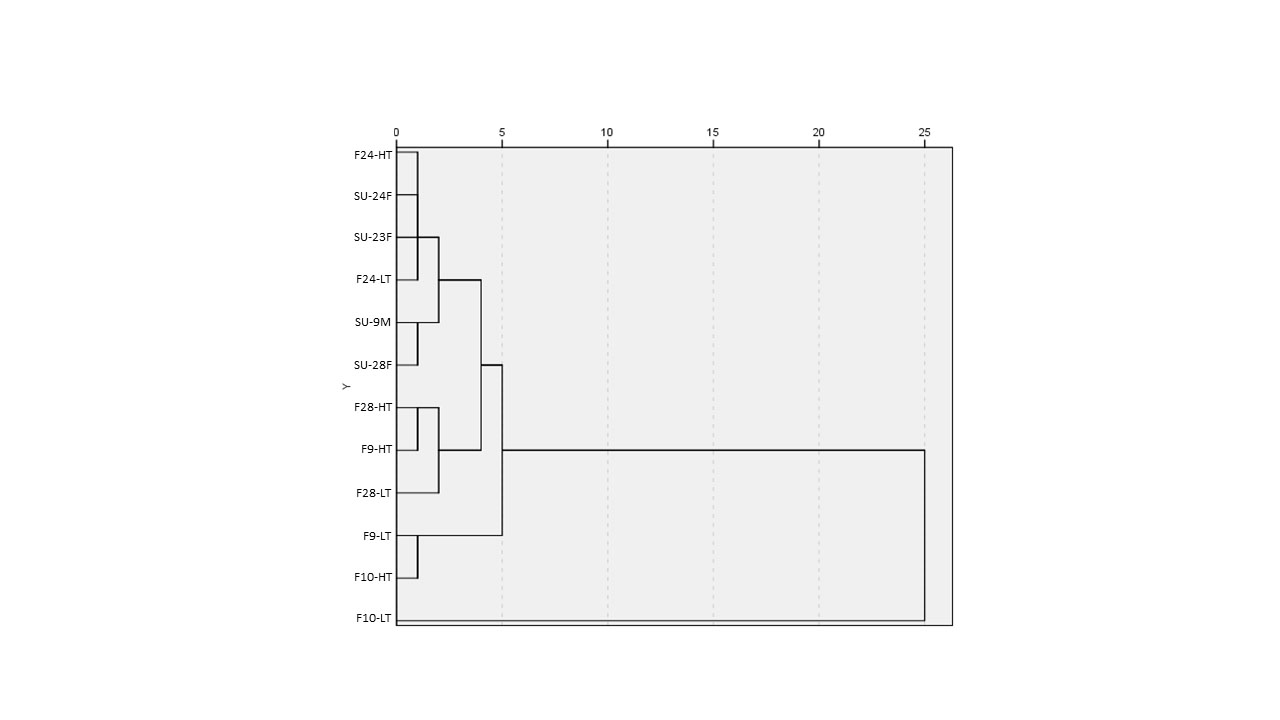


**Figure S5.** Hierarchical sample grouping tree calculated by Euclidean squared distance method adjusting the OTUs (at a 0.03 distance) obtained.


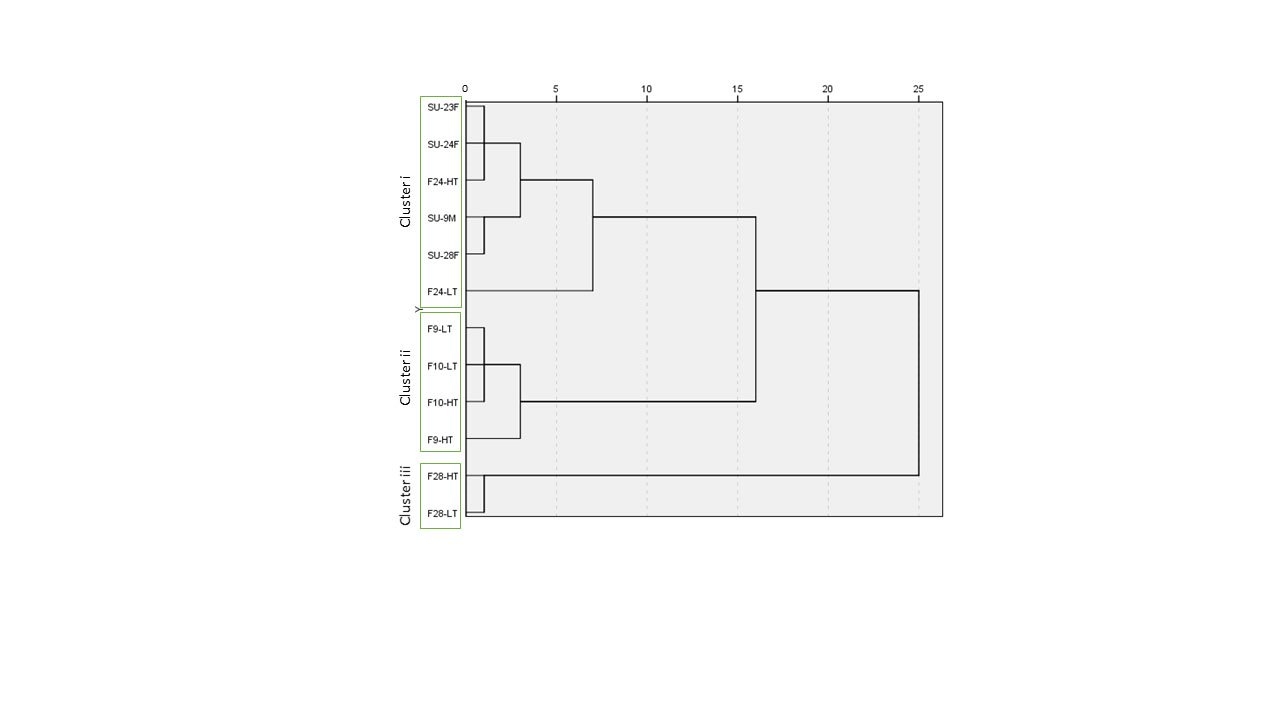


**Figure S6.** Relationships among bacterial populations in the different samples. Bacterial populations were clustered in a hierarchical dendrogram by Euclidean squared distance method calculated using SPSS.  Relative proportion of OTUs (at a 0.03 distance) among the samples was used.
